# A Meta-Analysis of the Impact of Resveratrol Supplementation on Markers of Renal Function and Blood Pressure in Type 2 Diabetic Patients on Hypoglycemic Therapy

**DOI:** 10.3390/molecules25235645

**Published:** 2020-11-30

**Authors:** Tawanda M. Nyambuya, Bongani B. Nkambule, Sithandiwe E. Mazibuko-Mbeje, Vuyolwethu Mxinwa, Kabelo Mokgalaboni, Patrick Orlando, Sonia Silvestri, Johan Louw, Luca Tiano, Phiwayinkosi V. Dludla

**Affiliations:** 1 School of Laboratory Medicine and Medical Sciences, College of Health Sciences, University of KwaZulu-Natal, Durban 4000, South Africa; mnyambuya@nust.na (T.M.N.); nkambuleb@ukzn.ac.za (B.B.N.); 218081787@stu.ukzn.ac.za (V.M.); 218086707@stu.ukzn.ac.za (K.M.); 2 Department of Health Sciences, Faculty of Health and Applied Sciences, Namibia University of Science and Technology, Windhoek 9000, Namibia; 3 Department of Biochemistry, Faculty of Natural and Agricultural Sciences, North-West University, Mmabatho 2745, South Africa; 36588296@nwu.ac.za; 4 Department of Life and Environmental Sciences, Polytechnic University of Marche, 60131 Ancona, Italy; p.orlando@univpm.it (P.O.); s.silvestri@univpm.it (S.S.); l.tiano@univpm.it (L.T.); 5 Biomedical Research and Innovation Platform, South African Medical Research Council, Tygerberg 7505, South Africa; johan.louw@mrc.ac.za; 6 Department of Biochemistry and Microbiology, University of Zululand, KwaDlangezwa 3880, South Africa

**Keywords:** resveratrol, polyphenols, diabetic complications, type 2 diabetes, metabolic syndrome, renal function, blood pressure, metformin, hypoglycemic therapy

## Abstract

Evidence on the beneficial effects of resveratrol supplementation on cardiovascular disease-related profiles in patients with type 2 diabetes (T2D) is conflicting, while its impact on renal function and blood pressure measurements remains to be established in these patients. The current meta-analysis included randomized controlled trials (RCTs) reporting on the impact of resveratrol supplementation on markers of renal function and blood pressure in patients with T2D on hypoglycemic medication. Electronic databases such as MEDLINE, Cochrane Library, Scopus, and EMBASE were searched for eligible studies from inception up to June 2020. The random and fixed effects model was used in the meta-analysis. A total of five RCTs met the inclusion criteria and involved 388 participants with T2D. Notably, most of the participants were on metformin therapy, or metformin in combination with other hypoglycemic drugs such as insulin and glibenclamide. Pooled estimates showed that resveratrol supplementation in patients with T2D lowered the levels of fasting glucose (SMD: −0.06 [95% CI: −0.24, 0.12]; *I*^2^ = 4%, p = 0.39) and insulin (SMD: −0.08 [95% CI: −0.50, 0.34], *I*^2^ = 73%, p = 0.002) when compared to those on placebo. In addition, supplementation significantly lowered systolic blood pressure (SMD: −5.77 [95% CI: −8.61, −2.93], *I*^2^ = 66%, p = 0.02) in these patients. Although resveratrol supplementation did not affect creatinine or urea levels, it reduced the total protein content (SMD: −0.19 [95% CI: −0.36, −0.02]; *I*^2^ = 91%, p = 0.001). In all, resveratrol supplementation in hypoglycemic therapy improves glucose control and lowers blood pressure; however, additional evidence is necessary to confirm its effect on renal function in patients with T2D.

## 1. Introduction

Noncommunicable diseases remain the leading cause of death worldwide [1,2]. Moreover, metabolic-related complications such as diabetes mellitus are known to play a key role in accelerating the global mortality rate [1,3,4]. As of 2017, it was estimated that approximately 415 million adults worldwide had diabetes, and it was predicted that this number will escalate and reach 642 million by the year 2040 [1]. Despite being one of the most investigated metabolic complications [5,6], diabetes continues to aggressively affect patients’ quality of life. For instance, it is now established that diabetes can adversely affect various essential organs of the body such as nerves, liver, and kidneys as well as the cardiovascular system, resulting in the development of neuropathy, retinopathy, nephropathy, and cardiovascular disease (CVD) [1,2,7,8]. Chronic hyperglycemia and insulin resistance are the hallmarks of the poor glucose control that drives the pathogenesis of type 2 diabetes (T2D). Notably, dyslipidemia, oxidative stress and low-grade inflammation are implicated in disease progression.

Increased understanding of the pathophysiological mechanisms involved in the progression of diabetes has paved the way for the exploration of long-term effective therapeutic strategies [9,10,11]. Thus far, metformin (PubChem CID: 4091) has been used as the drug of choice for the treatment of T2D [12,13]. In addition to acting in the gut to enhance glucagon-like peptide-1 secretion [14], metformin can block hepatic mitochondrial respiratory chain, and in the process, activate 5’ AMP-activated protein kinase (AMPK), leading to improved insulin sensitivity and reduced gluconeogenesis [15]. Although such beneficial effects of metformin are acknowledged, there is an increasing urge to understand the long-term efficacy of this anti-hyperglycemic drug in the management of diabetes and its associated complications. As a result, recent studies have explored its combinational use with some dietary supplements and nutraceuticals that are known to possess anti-hyperglycemic and antioxidative effects [16,17,18,19].

A prime example is resveratrol (PubChem CID: 445154), a natural phenol present in several food sources such as grapes and red wine [20]. Notably, resveratrol has been reported to have more protective effects over metformin in diabetes-associated complications in preclinical settings [21,22,23]. This has led to the initiation of several randomized controlled trials (RCTs) assessing the impact of resveratrol supplementation in patients with T2D on metformin therapy [24,25,26,27]. Several reviews have evaluated information on the beneficial effects of resveratrol against diabetic complications [28,29]; however, its combinational use with hypoglycemic drugs remains elusive. Therefore, the current meta-analysis aimed to assess whether resveratrol supplementation improves basic metabolic parameters in patients with T2D on hypoglycemic medication. Furthermore, we aim to evaluate whether resveratrol supplementation improves measurable outcomes linked with blood pressure and renal function in these patients, since hypertension and kidney dysfunction are common morbidities in a diabetic state [30,31].

## 2. Methods

This meta-analysis was prepared in accordance with the preferred reporting items for systematic reviews and meta-analysis (PRISMA) guidelines (Supplementary File). Although this meta-analysis has no registered protocol, all authors designed and agreed upon the procedure to be followed before conducting this study. In addition, we searched the international prospective register of systematic reviews (PROSPERO) database to ensure that there is no registered systematic review or meta-analysis that is investigating a similar topic.

This meta-analysis was performed to answer the following questions:

Question 1: Does resveratrol supplementation improve glucose control and blood pressure in patients with T2D on hypoglycemic therapies?

Question 2: What is the effect of resveratrol supplementation on renal function in individuals with T2D?

Eligibility criteria

- We included studies that met the following criteria:

Participants

- Adult patients with T2D.

Intervention

- Treatment intervention involved resveratrol supplementation in patients with T2D on metformin-based hypoglycemic therapies.

Comparator

- Patients with T2D on hypoglycemic therapy who did not receive resveratrol supplementation.

Outcome

- The primary outcome of this meta-analysis was glucose control, whilst the secondary outcome was renal function.

### 2.1. Search Strategy and Selection

A systematic search without any language restrictions was conducted by two independent reviewers (TMN and PVD) on the MEDLINE, Cochrane Library and EMBASE electronic databases from inception up to June 2020. The search strategy was adapted to the corresponding database to retrieve relevant studies using keywords and medical subjects heading (MeSH) terms “resveratrol”, “metformin”, “diabetes mellitus”, “metabolic syndrome”, and their corresponding synonyms and related words/phrases. In this meta-analysis, we included both randomized and non-randomized control trials that reported on the impact of resveratrol supplementation on glucose control, renal function and blood pressure in T2D patients on hypoglycemic therapies. We excluded observational studies, animal studies, reviews, editorials, books and letters to the editor.

### 2.2. Data Extraction

Two investigators (TMN and PVD) independently extracted study-level data using a pre-defined extraction data sheet. Data items included study; author’s name and year of publication; average age of patients; treatment dosages and intervention period; main findings; and outcome measures. Fasting blood glucose, glycated hemoglobin (Hb1Ac) and insulin were the extracted effect measures of the primary outcome, whereas systolic and diastolic blood pressure, creatinine, uric acid, and total protein were extracted as effect measures of the secondary outcome. In cases of disagreements regarding the extracted data items, a third investigator (BBN) was consulted for arbitration. EndNote version 10 (Clarivate Analytics, Philadelphia, PA, USA) was used to manage the extracted information and also to eliminate any duplicates.

### 2.3. Risk of Bias and Quality of Evidence assessment

The modified Downs and Black checklist, suitable for both randomized and non-randomized studies [32], was used to assess the risk of bias of the included studies. The checklist comprises four domains, namely reporting, external validity, internal validity and selection bias. Two investigators (VM and KM) independently assessed the included studies and rated them as poor if the score was < 12 points, fair if 13–18 points, good if 19–23 points, and excellent if the score was 24–27. Any discrepancies were resolved by consulting the third investigator (TMN), as previously reported [33].

### 2.4. Statistical Analysis

The RevMan software (version 5.0; Cochrane Collaboration, Oxford, UK) was used to conduct the meta-analysis and statistical analyses. Pearson’s chi-squared test (Chi^2^) and Higgin’s *I*^2^ statistics were used to assess statistical heterogeneity [34]. The fixed effects or random-effects model was used depending on the levels of statistical heterogeneity. Cohen’s method was used to interpret the effect sizes, whereby 0.2, 0.5 and 0.8 were equated to small, medium and large, respectively [35]. A subgroup analysis based on treatment dosages was performed to explore unexplained sources of heterogeneity. Interrater reliability was evaluated using Cohen’s kappa [36] and publication bias was assessed using visual inspection of funnel plots.

## 3. Results

### 3.1. Study Selection

**Figure 1:**
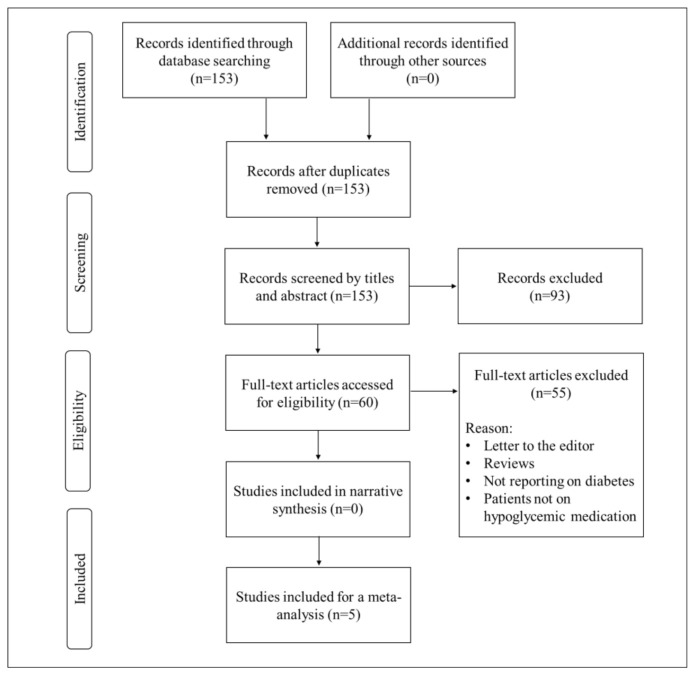
An overview of the flow diagram showing study inclusion.

We identified 153 citations through a combined systematic search of the literature. A total of 93 studies were excluded at the title and abstract stage. As a result, 60 studies were eligible for full-text screening, and a sum of 54 citations were excluded because they were letters to the editor, reviews, or not relevant to the topic of interest. One of the remaining studies was excluded (Bhatt et al., 2013) from the statistical analysis because it was a duplication of the population previously reported (Bhatt et al., 2012). Subsequently, five studies met the inclusion criteria and were included in the meta-analysis (Figure 1).

### 3.2. Study Characteristics

All included studies were RCTs published in peer-reviewed journals between 2012 and 2018, and were from five countries, namely India, Iran, Italy, The Netherlands, and Singapore (Table 1). Briefly, the meta-analysis comprised of a total of 388 participants, of which 56% were on resveratrol supplementation and 44% on a placebo. The mean age of participants was 59 years. Two of the included RCTs [24,37] reported the use of low dose (<500 mg/day) resveratrol, one study reported the use of high dose (>500 mg/day) [26], whilst the remaining study reported both low and high doses [38]. All hypoglycemic therapies in the included studies involved the use of metformin as a monotherapy or in combination with other glucose lowering drugs such as glibenclamide, sulfonylurea, and insulin. Detailed characteristic features of the included studies are presented in Table 1.

### 3.3. Risk of Bias Assessment and Publication Bias

The overall median score range of the included RCTs was 21.5 (17–24), with one study scored as fair (17 points), three scored as good (20–23 points), and two as excellent (24 points) (Supplementary File). The included studies had a low risk of reporting and selection bias with a median score range of 9.5 (9–10) out of a possible score of 10 (overall agreement 83.33, kappa = 0.67) and 5.3 (4–6) out of a possible score of 6 (overall agreement 90%, kappa = 0.80), respectively. In addition, the included studies scored high in internal validity with a median score of 4.3 (2–6) out of a possible score of 7 (overall agreement 80.95, kappa = 0.62). However, the studies had poor external validity with a median score of 2.3 (2–3) out of a possible score of 3 (overall agreement 66.67, kappa = 0.33). Therefore, caution needs to be taken when interpreting the findings outside this study population.

### 3.4. Impact of Resveratrol Supplementation on Basic Metabolic Parameters in T2D Patients on Hypoglycemic Medication

**Figure 2:**
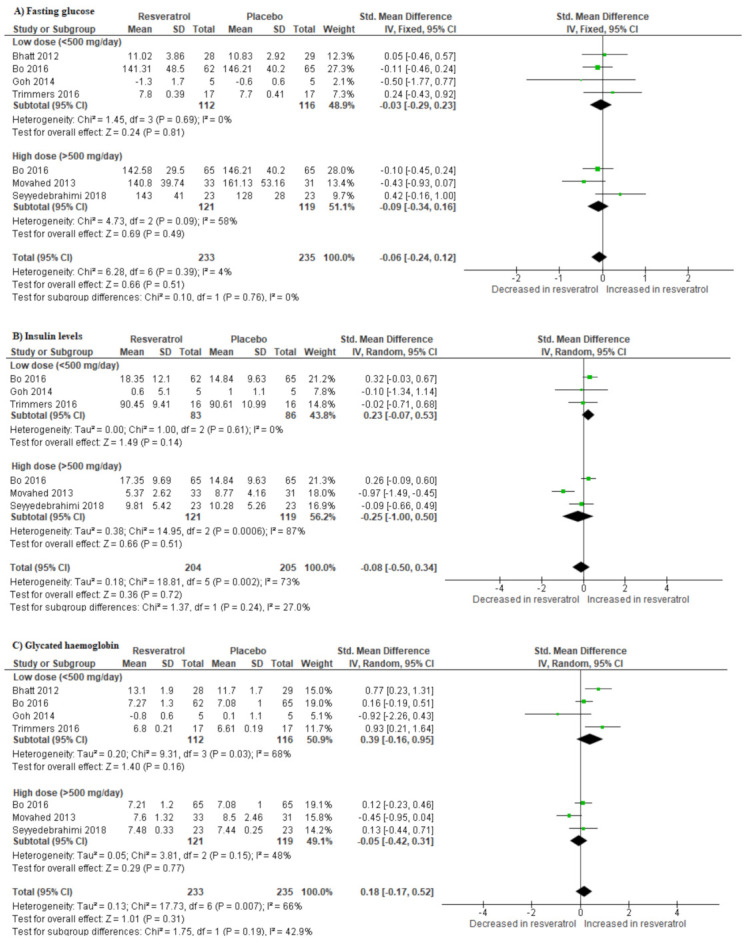
The dose-dependent effect of resveratrol supplementation on metabolic parameters, measured by changes in fasting glucose (**A**), insulin levels (**B**), and glycated hemoglobin (Hb1Ac; (**C**)) in patients with type 2 diabetes on hypoglycemic medication.

A total of four studies reported on the effect of resveratrol supplementation on glucose control. Individuals with T2D on resveratrol supplements had lower fasting glucose levels (SMD: −0.06 [95% CI: −0.24, 0.12]; *I*^2^ = 4%, p = 0.39) (Figure 2A) and insulin (SMD: −0.08 [95% CI: −0.50, 0.34], *I*^2^ = 73%, p = 0.002), respectively, when compared to those on placebo (Figure 2B). However, resveratrol supplementation did not affect the levels of Hb1Ac in comparison to placebo (SMD: 0.18 [95% CI: −0.17, 0.52]; *I*^2^ = 66%, p = 0.007) (Figure 2C).

To investigate the potential sources of substantial levels of statistical heterogeneity, we conducted a subgroup analysis based on the dosage of resveratrol, since the test for subgroup differences was significant (p > 0.08) [41]. Notably, both low and high dosages of resveratrol decreased the level of fasting blood glucose, whilst only the high dose was associated with a reduction in insulin (SMD: −0.25 [95% CI: −1.00, 0.50]; *I*^2^ = 87%, p = 0.0006) and Hb1Ac (SMD: −0.05 [95% CI: −0.42, 0.31]; *I*^2^ = 48%, p = 0.15) levels (Figure 2).

### 3.5. Impact of Resveratrol Supplementation on Markers of Renal Function in T2D Patients on Hypoglycemic Therapy

**Figure 3:**
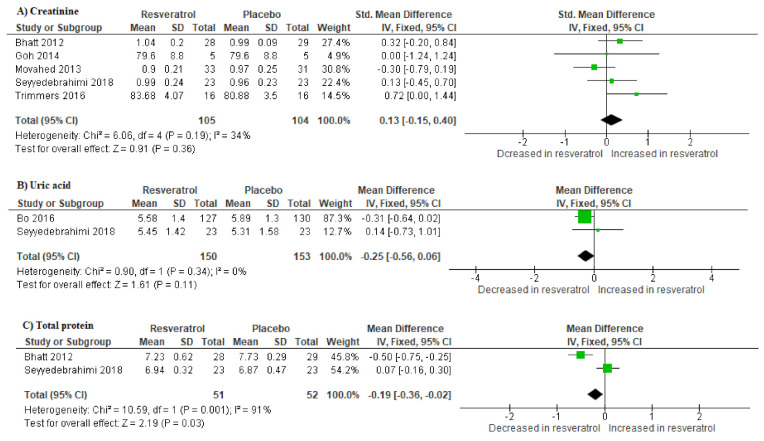
The impact of resveratrol supplementation on markers of renal function denoted by the levels of creatinine (**A**), uric acid (**B**) and total protein (**C**) in patients with type 2 diabetes on hypoglycemic therapy.

A total of six studies reported on the effect of resveratrol supplementation on renal function compared to placebo. Individuals with T2D on resveratrol supplements had higher levels of creatinine (SMD: 0.13 [95% CI: −0.15, 0.40]; *I*^2^ = 34%, p = 0.19) (Figure 3A); decreased levels of uric acid (SMD: −0.25 [95% CI:−0.56, 0.06]; *I*^2^ = 0%, p = 0.34) (Figure 3B); and total protein (SMD: −0.19 [95% CI: −0.36, −0.02], *I*^2^ = 91%, p = 0.001) (Figure 3C) when compared to the placebo (Figure 3).

### 3.6. Resveratrol Supplementation Lowers Blood Pressure in T2D Patients on Hypoglycemic Drugs

**Figure 4:**
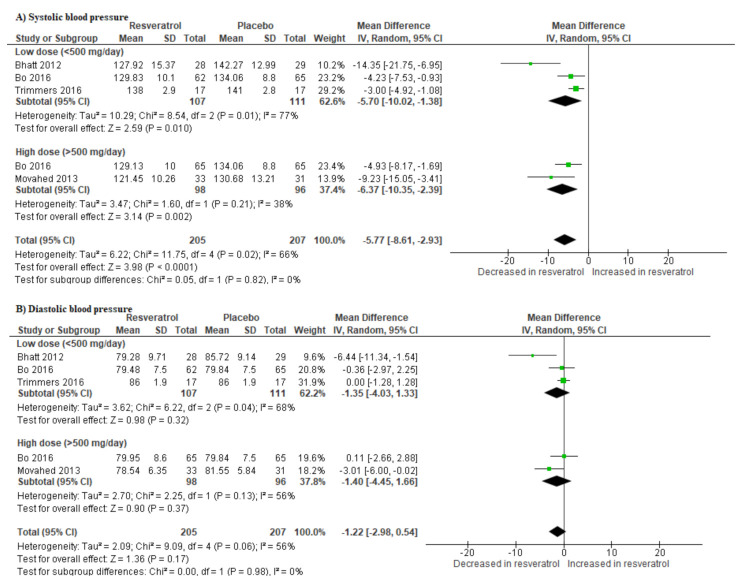
The impact of resveratrol supplementation on the systolic (**A**) and diastolic blood (**B**) pressures of T2D patients on hypoglycemic medication.

Individuals with T2D receiving resveratrol supplementation had significantly lower systolic blood pressure when compared to those receiving a placebo (SMD: −5.77 [95% CI: −8.61, −2.93]; *I*^2^ = 0%, p = 0.02) (Figure 4A). Likewise, their diastolic blood pressure was lower in comparison to the placebo group (SMD: −1.22 [95% CI: −2.98, 0.54]; *I*^2^ = 56%, p = 0.06) (Figure 4B).

## 4. Discussion

Dietary supplements and functional food ingredients are increasingly being explored for their diverse therapeutic capabilities [18,42,43]. As such, several dietary supplements have gained special interest due to their envisaged ameliorative effects against lifestyle diseases such as T2D and cardiovascular complications [17,18,19,23,24,29]. Consequently, a very large population of patients with diabetes, particularly in developed countries, are now taking dietary supplements to enhance the efficacy of their medication [44,45]. Therefore, it remains crucial to understand the beneficial effects of dietary supplements such as resveratrol, especially when taken together with underlying glucose-lowering drugs in T2D patients. The current meta-analysis is the first to explore and critically discuss the impact of resveratrol supplementation in patients with T2D on metformin therapy.

Through an extensive literature search, we identified six RCTs [24,26,37,38,39,40] assessing the impact of resveratrol against diabetes-associated complications in patients on hypoglycemic medication. In addition to improving blood glucose control, results from the pooled estimates showed that resveratrol supplementation could significantly reduce CVD-risk by lowering blood pressure. Notably, while other meta-analyses [28,46,47,48] have already shown that resveratrol supplementation is beneficial in those with T2D or blood pressure, from the current findings, it is evident that this natural compound does not induce any adverse effects that may relate to drug–drug interaction or severe hypoglycemia. Most importantly, these results were independent of dose selection, as it was evident that doses less or more than 500 mg/day were equally effective in improving blood glucose control and lowering blood pressure. Thus, although precautions have been previously discussed in relation to resveratrol interacting with various drug-metabolizing enzymes such as cytochrome P450 (CYP) [49], this does not interfere with its efficacy in patients with T2D on hypoglycemic medication.

Furthermore, since hypertension is closely associated with renal failure in patients with diabetes [50], we further assessed whether improved blood pressure correlated with any regulation in markers of renal function. Interestingly, although resveratrol supplementation improved blood pressure in patients with T2D, this effect was independent of its effects on renal function. Although studies included within the meta-analysis were very limited, the current results showed that resveratrol did not affect creatinine or uric acid levels. The levels of total protein were significantly reduced; however, this occurring independent of other markers (creatinine and uric acid) does not indicate any compelling support for the positive effects of resveratrol supplementation in improving renal function. Although evidence from clinical settings is scarce, preclinical studies have suggested that resveratrol may have a protective effect against renal diseases, through attenuation of oxidative stress and the activation of NAD-dependent deacetylase sirtuin-1 (SIRT1), as reviewed elsewhere [51,52,53]. Thus, additional long-term clinical studies are required to directly assess the combined impact of resveratrol and metformin in managing T2D and its related complications.

The major weakness of this study was the differences in treatment dosages in the included studies. This could have influenced the high levels of unexplained statistical heterogeneity and differences in the reported effects of resveratrol supplementation. Nonetheless, our study has a unique strength, that is, it is the first to assess the effect of resveratrol supplementation on markers of metabolic syndrome in T2D patients on hypoglycemia medication. Moreover, the methods used by the two independent reviewers in the study selection and data extraction processes as well as risk of bias and quality of evidence assessment were robust, as indicated by high interrater agreement. In addition, the quality of evidence was high due to the designs of the included studies and low risk of bias. Lastly, since the included studies scored high in the external validity, the findings of this study can be generalized and are applicable to the rest of the world.

## 5. Conclusions

Resveratrol is a non-flavonoid polyphenol that naturally occurs as phytoalexin and has been extensively studied in animal models and in diabetic humans [20,43,47]. This explains the general interest in understanding the therapeutic benefits of dietary supplements such as resveratrol in improving human health, especially their synergistic effects when used with established oral glucose-lowering drugs like metformin. In fact, while metformin remains a drug of choice to treat T2D, the continued rise in diabetes-related deaths warrants further investigation into novel and improved therapies to prolong the lives of diabetic patients. Consistent with the current study, a recent meta-analysis already supported the safety and beneficial effects of this natural polyphenol in diabetic patients, and its possible use as an active compound to promote cardiovascular health, mostly when used in a high daily dose (≥300 mg/day) [47]. Although emerging clinical studies support the beneficial effects of resveratrol to ameliorate diabetes-linked abnormalities, less is currently known as to how this dietary compound benefits patients on hypoglycemic medication, predominantly metformin therapy. The data summarized in this meta-analysis support the beneficial effects of resveratrol in improving blood glucose control and lowering blood pressure, with additional evidence required to confirm its modulation of renal function in T2D patients on metformin therapy.

## Figures and Tables

**Table 1:** Characteristics of studies reporting on resveratrol supplementation in patients with type 2 diabetes on hypoglycemic therapy.

Author, Year	Country	Sample Size Average Age (% Male)	Experimental Model, Dose Used, and Intervention Period	Experimental Outcome and Proposed Mechanism
Bhatt et al., 2012 [24]	India	Total = 57 (*n* = 29 controls/*n* = 28 intervention) Average age = 57 (37%)	Type 2 diabetic (T2D) patients, on minimum of 6 months of ongoing oral hypoglycemic treatment (metformin and/or glibenclamide), received resveratrol at 250 mg/daily for 3 months	Supplementation with resveratrol significantly improved the mean glycated hemoglobin (HbA1c), systolic blood pressure, and total protein in T2D. No significant changes in body weight and high-density lipoprotein and low-density lipoprotein cholesterols
Movahed et al., 2013 [26]	Iran	Total = 66 (*n* = 33 controls/*n* = 33 intervention) Average age = 52 (50%)	T2D patients, on metformin therapy received resveratrol at a dose 1 g/day for 1½ months and a control group which received placebo tablets	Resveratrol treatment significantly decreased systolic blood pressure, fasting blood glucose, hemoglobin A1c, insulin, and insulin resistance, while high-density lipoprotein was significantly increased when compared to their baseline levels. Liver and kidney function markers were unchanged in the intervention group
Goh et al., 2014 [39]	Singapore	Total = 10 (*n* = 5 controls/*n* = 5 intervention) Average = 56 (100%)	T2D patients, oral hypoglycemic agents, received resveratrol at a dose of 3 g or placebo for 3 months	Resveratrol regulated energy expenditure through increased skeletal muscle NAD-dependent deacetylase sirtuin-1 (SIRT1) and 5′ AMP-activated protein kinase (AMPK) expression
Bo et al., 2016 [38]	Italy	Total = 192 (*n* = 62 control/*n* = 130 intervention) Average age = 65 (66%)	T2D patients received resveratrol supplementation at two different dosages (500 and 40 mg/day) for 6 months, of which 67.7% were on metformin	Treatment did not affect body weight, body mass index, waist circumference, and values of arterial blood pressure, fasting glucose, glycated hemoglobin, insulin, C-peptide, free fatty acids, liver transaminases, uric acid, adiponectin, and interleukin-6, in both the Resv500 and Resv40 arms vs. placebo. Total cholesterol and triglycerides slightly increased with resveratrol treatment
Timmers et al., 2016 [37]	The Netherlands	Total = 17 Average age = 64 (100%)	T2D patients treated with placebo and 150 mg/day resveratrol and average metformin dose of 2188 mg/day in a randomized double-blind crossover study for 1 month	Hepatic and peripheral insulin sensitivity were not affected by resveratrol treatment. Resveratrol also significantly improved ex vivo mitochondrial function
Seyyedebrahimi et al., 2018 [40]	Iran	Total = 46 (*n* = 23 controls/*n* = 23 intervention) Average age = 57 (86%)	T2D patients received resveratrol supplementation or placebo at a dose of 800 mg/day for 2 months	Resveratrol reduced plasma protein carbonyl content and significantly increased plasma total antioxidant capacity and total thiol content. However, it had no effect on metabolic parameters

## Supplementary Materials

Supplementary file: Preferred Reporting Items for Systematic Reviews and Meta-Analysis (PRISMA) Checklist.

## Abbreviations

AMPK	AMP-activated protein kinase
CVDs	Cardiovascular diseases
FPG	Fasting plasma glucose
GRADE	Grading of Recommendations Assessment Development and Evaluation
HbA1c	Glycated hemoglobin
PRISMA	Preferred Reporting Items for Systematic reviews and Meta-Analysis
RCT	Randomized controlled trials
SMD	Standardized mean difference
T2D	Type 2 diabetes
